# isONform: reference-free transcriptome reconstruction from Oxford Nanopore data

**DOI:** 10.1093/bioinformatics/btad264

**Published:** 2023-06-30

**Authors:** Alexander J Petri, Kristoffer Sahlin

**Affiliations:** Department of Mathematics, Science for Life Laboratory, Stockholm University, Stockholm 106 91, Sweden; Department of Mathematics, Science for Life Laboratory, Stockholm University, Stockholm 106 91, Sweden

## Abstract

**Motivation:**

With advances in long-read transcriptome sequencing, we can now fully sequence transcripts, which greatly improves our ability to study transcription processes. A popular long-read transcriptome sequencing technique is Oxford Nanopore Technologies (ONT), which through its cost-effective sequencing and high throughput, has the potential to characterize the transcriptome in a cell. However, due to transcript variability and sequencing errors, long cDNA reads need substantial bioinformatic processing to produce a set of isoform predictions from the reads. Several genome and annotation-based methods exist to produce transcript predictions. However, such methods require high-quality genomes and annotations and are limited by the accuracy of long-read splice aligners. In addition, gene families with high heterogeneity may not be well represented by a reference genome and would benefit from reference-free analysis. Reference-free methods to predict transcripts from ONT, such as RATTLE, exist, but their sensitivity is not comparable to reference-based approaches.

**Results:**

We present isONform, a high-sensitivity algorithm to construct isoforms from ONT cDNA sequencing data. The algorithm is based on iterative bubble popping on gene graphs built from fuzzy seeds from the reads. Using simulated, synthetic, and biological ONT cDNA data, we show that isONform has substantially higher sensitivity than RATTLE albeit with some loss in precision. On biological data, we show that isONform’s predictions have substantially higher consistency with the annotation-based method StringTie2 compared with RATTLE. We believe isONform can be used both for isoform construction for organisms without well-annotated genomes and as an orthogonal method to verify predictions of reference-based methods.

**Availability and implementation:**

https://github.com/aljpetri/isONform

## 1 Introduction

Long-read sequencing techniques such as Oxford Nanopore Technologies (ONT) and Pacific Biosciences (PacBio) can produce reads covering the majority of transcripts from end-to-end. Such sequencing techniques have proven to enable a better understanding of the transcriptional landscape of cells (Bayega et al. 2018; Byrne et al. 2019; Cole et al. 2020). However, while long-read methods omit the need for transcript assembly as needed with short reads, transcript 3′ and 5′ variability, RNA degradation, long-read sequencing error profiles, and other sequencing artifacts inhibit downstream analysis of long-read data. Particularly, recovering the actual isoforms from long-read transcriptomic datasets has proven difficult, with studies observing thousands of low-quality and potentially spurious transcript predictions (Hoang et al. 2017; Kuo et al. 2020).

### 1.1 Genome- and annotation-based methods

Due to the noise in these datasets, a common approach to predict transcripts is to align the transcriptomic reads to a reference genome using a long-read splice aligner such as minimap2 (Li 2018), deSALT (Liu et al. 2019), or uLTRA (Sahlin and Mäkinen 2021). Several tools for reconstructing transcripts from long-read splice alignments have been proposed (Kovaka et al. 2019; Tung et al. 2019; Tang et al. 2020; Kuo et al. 2020; Wyman et al. 2020; Holmqvist et al. 2021; Orabi et al. 2022; Chen et al. 2022; Volden et al. 2022; Prjibelski et al. 2023). These tools typically predict transcripts by requiring both alignments and transcript annotations (Kovaka et al. 2019; Tung et al. 2019; Tang et al. 2020; Kuo et al. 2020; Wyman et al. 2020; Holmqvist et al. 2021; Chen et al. 2022; Volden et al. 2022), or requiring alignments but with the ability to predict transcripts outside annotations (Orabi et al. 2022; Prjibelski et al. 2023). A thorough community-effort benchmarking of many of these tools using several different sequencing techniques and genomes has recently been performed as part of the LRGASP challenge (Pardo-Palacios et al. 2021), and preliminary results of challenge 1 (LRGASP 2022) show a large discrepancy between methods in isoform detection, even for organisms with high-quality reference genomes such as mouse and human.

Larger discrepancies in predicted transcripts between methods can be explained by the different algorithms they use, as well as error levels and artifacts of the sequencing techniques. However, there are also inherent limitations with predicting transcripts from alignments to a linear reference genome. Such limitations include predicting transcripts from fused genes, gene copies, or exons that are not present in the linear genome or shortcomings in splice alignment, which the predictions fundamentally rely on. For example, in the study by Sahlin and Mäkinen (2021), we showed that long-read splice alignment methods have limitations such as (i) small exons not being detected by read aligners, (ii) overfitting to canonical splice sites, and (iii) not aligning over long introns. Additionally, relying on a reference genome and reference annotation limits the methods to organisms and genes for which reference annotations exist.

### 1.2 Genome- and annotation-agnostic methods

By not relying on high quality genome assemblies and annotations, reference-free approaches are important for research of lesser known organisms/gene families. In addition, they can overcome the splice-alignment limitations and the bias introduced by linear reference genomes. So far, relatively few methods have been proposed for reference-free transcript predictions. Some tools have been proposed for PacBio sequencing such as isoseq3 (Gordon et al. 2015) and IsoCon (Sahlin et al. 2018) but are limited to PacBio data and IsoCon is only applicable to targeted sequencing. While there exists recent unpublished work (Nip et al. 2022), the only published tool for reference-free transcriptome reconstruction of ONT reads is RATTLE (de la Rubia et al. 2022). However, as we show in our study, RATTLE has low transcript prediction sensitivity and misses many transcripts present in data. Some challenges of reference-free transcript reconstruction are highly variable abundance, alternative splicing, as well as long-read error profiles.

### 1.3 Our contribution

We have previously demonstrated algorithms for clustering and error correction of long transcriptomic reads through isONclust (Sahlin and Medvedev 2020) and isONcorrect (Sahlin and Medvedev 2021). We here introduce an algorithm and its implementation, isONform, for the final step of this pipeline to produce transcript predictions. IsONform builds a graph based on paired-minimizer seeds, that, similarly to minimizer-based genome assembly approaches (Rautiainen and Marschall 2021; Ekim et al. 2021), aims to sparsely represent the sequence information in the data. IsONform then employs iterative bubble popping on the graph to remove errors while keeping exon differences. When no poppable bubbles exist, isoform predictions can be traced from the graph by following paths of full-length reads. A fundamental difference between our isON pipeline (isONclust, isONcorrect, and isONform) and RATTLE’s pipeline (cluster, error correct, and polish) is that the isON pipeline works with clusters of reads from a gene, while RATTLE clusters by transcript. This allows isON’s error correction and consensus prediction (isONform) to leverage shared exons among splice variants, thus, the potential to recover more transcripts with higher base-level accuracy compared with predictions by RATTLE. However, to benefit from shared exons in different transcripts, it also requires a more sophisticated consensus generation algorithm able to separate isoforms at the consensus calling step, compared with RATTLE, which only does base-level polishing per transcript in the consensus (polishing) step. Using simulated and synthetic reads, we show that isONform recovers substantially more isoforms than RATTLE. On biological data, we show that isONform has much higher consistency with the reference-based tool StringTie2 compared with RATTLE. We believe that isONform (integrated into our isON pipeline) has two primary use cases. First, it can be used for isoform construction for organisms without well-annotated genomes. Second, it can be used as an orthogonal method to verify consistency in reference-based prediction since systematic limitations exist, as described in Section 1.1.

## 2 Materials and methods

In Section 2.1, we give a high level overview of the algorithm. In Section 2.2, we introduce notation and formally define minimizer pairs and the graph structure that isONform uses. Finally, we describe the algorithm in depth in Section 2.3 and implementation details in Section 2.4.

### 2.1 Algorithm overview

**Figure 1.**
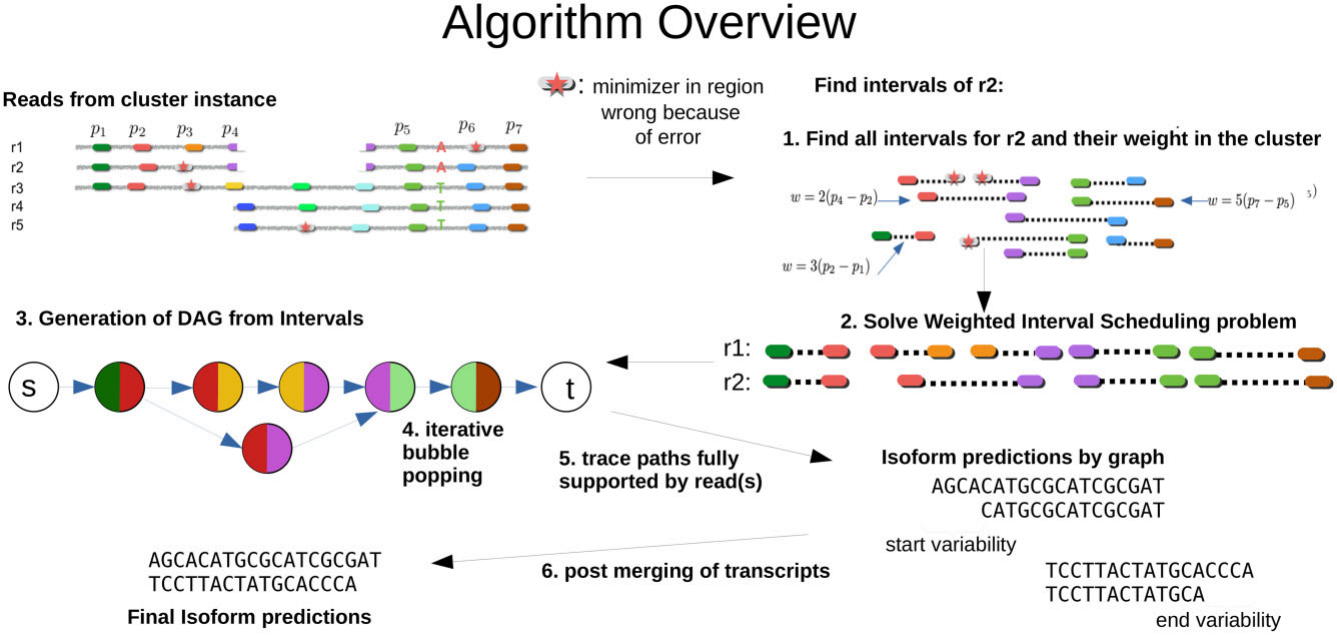
Overview of the isONform algorithm. IsONform takes as input clustered and error-corrected reads generated with isONclust and isONcorrect, respectively. The example illustrates a cluster consisting of five reads (*r*_1_ to *r*_5_) from three isoforms, where *p* denotes positions of minimizers on *r*_1_. isONform uses all minimizer pairs from minimizers at distance *x_min_* to *x_max_*. The figure shows an example of 10 minimizer pairs on *r*_2_. Each minimizer pair is assigned a weight formed from the length of the minimizer pairs and its abundance in the cluster. The instance is then sent to a weighted interval scheduler, which is used to find the set of NOMPs. In our example, we find five segments for *r*_1_ while the scheduler finds four instances for *r*_2_. A DAG is built up by the NOMPs, and we add a global source node *s* as well as a global sink node *t* (step 3). An iterative bubble-popping approach is employed to merge similar paths in the graph (step 4). This reduces the number of paths supported by reads (step 5). We finally perform postmerging of the transcripts due to 3′ and 5′ variability. For each isoform, a consensus sequence is generated from all reads appointed to it using the SPOA algorithm.

The input of our algorithm are clustered and error-corrected reads from isONclust (Sahlin and Medvedev 2020) and isONcorrect (Sahlin and Medvedev 2021). Each cluster represents reads from isoforms from a gene family and can be processed individually and in parallel with isONform. The output is a set of predicted transcripts in fastq format. We describe the algorithm for reads from a single cluster. In step 1, reads are partitioned into intervals using “minimizer pairs” (Sahlin and Medvedev 2021) as seeds. In step 2, a weighted interval scheduling (WIS) problem is solved to find a set of maximally weighted and nonoverlapping minimizer pairs (NOMPs) for each read as done in isONcorrect (Sahlin and Medvedev 2021). In step 3, the set of NOMPs is used to construct a directed acyclic graph (DAG) in which vertices represent the NOMPs and neighboring NOMPs are connected with an edge. In step 4, the graph is simplified by an iterative bubble-popping algorithm designed to remove errors and mutations between reads while keeping exon differences. In step 5, predicted isoforms are generated by finding supported paths through the graph. Finally, step 6 removes any redundant isoform predictions caused mainly by the larger 3′ and 5′ variability using pairwise alignment of predicted isoforms. The isoform predictions remaining after this step constitute the final predictions. An overview of the algorithm is shown in Fig. 1.

### 2.2 Preliminaries

isONform uses the same generation of minimizers and minimizer-pairs as described in isONcorrect (Sahlin and Medvedev 2021). We describe them here for completeness.

#### 2.2.1 Minimizers

Minimizers (Roberts et al. 2004) are subsampled *k*-mers. Let r∈R denote a string of nucleotides, which we refer to as “read.” We use r[i] to refer to the nucleotide located at the *i*-th position of *r*. Given two integers *k* and *w* such that 1≤k≤w≤|r|, the minimizer of *r* at position *p* is the lexicographically smallest k-mer *m* that starts inside the interval [p,p+w). We then say that *m* is a minimizer of the read *r*, or, alternatively, is a positional minimizer (m,p) of read *r*. Let *M*(*r*) be the set of positional minimizers of read *r*. In the following example, we use r=ACGGATCAC,k=2,w=4, yielding the set of positional minimizers M(r)=(AC,0),(CG,1),(AT,4),(AC,7).

#### 2.2.2 Minimizer pairs

Let *x_min_* and *x_max_* be two positive integer parameters. We let $W_{r}=\{((m_{i},p_{i}),(m_{j},p_{j}))\in M(r)\times M(r)|x_{\min}\leq p_{j}-p_{i}\leq x_{\max}\}$ be the ordered set (according to increasing *p_i_* then *p_j_*) of paired positional minimizers separated by at least *x_min_* and at most *x_max_* nucleotides in *r*. Similarly, we denote the sequence of paired minimizers as $\mathrm{Str}W_{r}=\{(m_{i},m_{j})|((m_{i},p_{i}),(m_{j},p_{j}))\in W_{r}\}$, i.e. *W_r_* with the positions omitted but duplicates retained. The above example with the following parameters *x_min_* = 2, *x_max_* = 4 therefore yields $W_{r}=((AC,0),(AT,4)),((CG,1),(AT,4)),((AT,4),(AC,7))$ and $\mathrm{str}W_{r}=(AC,AT),(CG,AT),(AT,AC)$. Given a set of reads *R*, we let *W* be the union of all *W_r_* for the reads in *R* and we let *StrW* be the union of all *StrW_r_*. Besides error correction (Sahlin and Medvedev 2021), minimizer pairs have also been used in genome assembly (Chin and Khalak 2019).

#### 2.2.3 Nonoverlapping minimizer pairs

For each read, isONform, similarly to isONcorrect, produces a subset of NOMPs from the set of minimizer pairs. The NOMPs are decided upon by the solution to a WIS problem. Specifically, the minimizer pairs span an “interval” on the read. The input to the WIS problem is a set of minimizer pairs (intervals) $I=I_{1},\ldots I_{n}$, where $I_{j}\in [a_{j},b_{j}],a_{j},b_{j}\in R$ and *a_j_* < *b_j_*. The number of reads supporting an interval *I_j_* (provided in *StrW*) gives a weight *w_j_* to *I_j_*. The solution to the WIS problem outputs a subset I′⊂I of nonoverlapping intervals for which the sum of weights is maximized. Our intervals in the solution I′ correspond to NOMPs that we index in the order they appear on the read, e.g. ${\text{NOMP}}_{j}^{r}$ is the *j*th NOMP in *r*. The WIS problem can be solved exactly by applying a dynamic programming algorithm that runs with O(n log n) time complexity, with *n* denoting the number of intervals (Kleinberg and Tardos 2006).

#### 2.2.4 Graphs of nonoverlapping minimizer pairs

We represent the NOMPs as vertices, where $v_{i}^{r}$ denotes the *i-*th NOMP on read *r*. We draw a directed edge $e_{ij}^{r}$ between two vertices $v_{i}^{r}$ and $v_{j}^{r}$ if $v_{i}^{r}$ and $v_{j}^{r}$ represent neighboring NOMPs on a read. This forms a directed graph G=(V,E), where we insert a global source and sink node *s* and *t*, respectively. We draw a directed edge (s,v′) from the global source to the first NOMP v′ on each read. Similarly, we draw a directed edge (v″,t) from the last minimizer pair v″ on each read to the global sink. A read spells a path from the global source to the sink.

Each node $v_{i}^{r}$ also contains information on the start and end position and the length (span) of the NOMP on read *r*. Furthermore, each edge $e_{i,i+1}^{r}$ has a length $l(e_{i,i+1}^{r})$ associated with it, representing the distance between the *i-*th and (*i* + 1)-th NOMP on *r*. Therefore, a path *p* in *G* that follows a read *r* has a corresponding length ($\left|p_{r}\right|$), start, and stop position on *r*. The graph is built up by iterating through the reads in order of appearance in the fastq file. A NOMP that is shared between reads will be represented by a single node. A NOMP is considered shared if it has the same minimizer pair and they do not have a difference in their pairwise distance larger than *δ* (parameter to isONform). However, isONform stores the length of each NOMP in the vertex. A detailed description of the graph construction is given in Section 2.3.1.

#### 2.2.5 Bubbles and multibubbles

**Figure 2.**
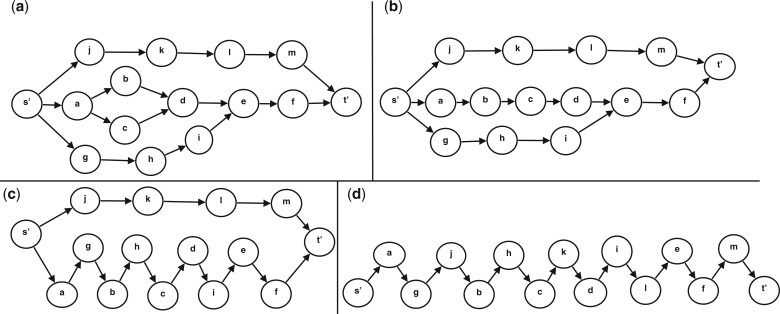
Bubble popping on a graph. (a) Our initial graph. There are three bubbles in this graph ({a, b, c, d},{s’,a, c, d, g, h, i, e}, and {s’,a, b, d, e, f, g, h, i, j, k, l, m, t’}). The bubble popping starts with the smallest bubble (fewest vertices) and continues until no more bubbles can be popped. (b) Bubble {a, b, c, d} was popped and its nodes linearized. (c) Bubble {s’,a, b, c, d, g, h, i, e} was popped and its nodes linearized. (d) The bubble in (c) could also be popped and we now have a single path from s′ to t′, representing a single isoform in the graph.

We define a bubble as a pair of paths with a shared start node *b_s_* and end node *b_e_* where the internal path nodes are disjoint. Note that nodes that are not part of the bubble can be reached from nodes belonging to the bubble. If the bubble additionally has at least one read supporting each path from *b_s_* to *b_e_* we denote it as “read-supported bubble.” We will detect and resolve what we refer to as read-supported multibubbles. An example of a multibubble structure is shown in Fig. 2a. Multibubbles have similar characteristics as superbubbles (Onodera et al. 2013) except that multibubbles allow nodes not part of the multibubble to be reachable from nodes in the multibubble and for several bubbles to start/end in one node, i.e. they do not fulfil the minimality and the matching criteria (Onodera et al. 2013). Let G=(V,E) a directed graph and (*s*, *t*) being an unordered pair of distinct vertices. Then, a multibubble is defined if it satisfies the following criteria:

- **Reachability**: *t* can be reached from *s.*
- **Acyclicity**: the subgraph induced by *U* is acyclic where *U* is the set of vertices as described above.

then we say that the subgraph in the description of the acyclicity condition forms a multibubble and *s*, *t* and U∖{s,t} are the multibubble’s source, sink and interior respectively.

### 2.3 Algorithm

Steps 1 and 2 consist of constructing minimizer pairs and solving the WIS problem to produce NOMPs as input to isONform. These steps are performed identically to isONcorrect.

#### 2.3.1 Step 3: graph construction

The graph is built up by iterating through the reads in order of appearance in the fastq file, and adding vertices and edges from the NOMPs produced in step 2. We want to keep the graph acyclic. Repetitive NOMPs within a read may cause a cycle. Also, since NOMPs are fuzzy seeds, they may have different lengths. Therefore, we want to identify larger length differences between NOMPs shared between reads. Finally, there can be gaps between neighboring NOMPs in reads. If two reads share two neighboring NOMPs, the gap between the two NOMPs may differ, indicating a structural difference between the reads. We handle those cases as follows.

**Preserving acyclicity:** For each new ${\text{NOMP}}_{i+1}^{r}$ to be added to *G*, if ${\text{NOMP}}_{i+1}^{r}$ is already found in the graph it may create a cycle if we add the edge $e_{i,i+1}^{r}$ connecting it to its predecessor. We can prevent *G* becoming cyclic by looking at the topological order of the graph. If $e_{i,i+1}^{r}$ would introduce a cycle, we instead add a new vertex $v_{i+1}^{r}$ to *G* representing ${\text{NOMP}}_{i+1}^{r}$.

**Differences in NOMP span:** For each ${\text{NOMP}}_{i+1}^{r}$ to be added to *G*, if the NOMP is already found in the graph, we check the length difference of ${\text{NOMP}}_{i+1}^{r}$ and the NOMP of the node existing in the graph. If the NOMP lengths differ by more than *δ* nucleotides, we add a new node. If a NOMP is already represented by two or more NOMPs, ${\text{NOMP}}_{i+1}^{r}$ is added to the vertex that has the NOMP with the closest distance to ${\text{NOMP}}_{i+1}^{r}$.

**Differences between NOMPs:** In a similar fashion to checking differences in NOMP span, if we find two reads *r* and r′ having the same neighboring NOMPs $\left({\text{NOMP}}_{i}^{r},{\text{NOMP}}_{i+1}^{r}\right)$ = $\left({\text{NOMP}}_{j}^{r\prime },{\text{NOMP}}_{j+1}^{r\prime }\right)$ but $|l(e_{i,i+1}^{r})-l(e_{j,j+1}^{r\prime })|>\delta$ then we create a separate vertex $v_{i+1}^{r\prime }$ for the second NOMP while adding the first NOMP to the vertex already in the graph.

#### 2.3.2 Step 4: iterative bubble popping

NOMPs are indel tolerant seeds, but sequencing errors and exon differences may result in local bubbles (Zerbino and Birney 2008) in *G*. It requires only a few bubbles to lead to many reads having unique paths through *G* (possibly exponentially increasing with the number of bubbles), which yields redundant consensus sequences. The purpose of the iterative bubble-popping step is to remove local bubbles in the graph caused by read errors and SNP differences while preserving differences at the exon level between reads.

**Overview:** At a high level, *G* may contain several multibubbles sharing vertices. We find disjoint “read-supported bubbles” that we can pop during each iteration of our iterative bubble popping. We start by analyzing the smallest bubbles (with respect to the number of vertices) and gradually test and pop bubbles until no more new bubbles can be detected in the graph. We test if a bubble is poppable by forming a consensus (using SPOA, Vaser et al. 2017) of the subsequences of reads corresponding to each path in the bubble. If such consensuses do not differ in length by more than *δ* and have sequence similarity higher than *α* (parameter to isONform), we pop the bubble. Otherwise, we save it as unpoppable. If a bubble can be popped, we use the relative distance information to reorder (or “linearize”) the vertices of the two paths. At each new iteration, the bubbles we previously investigated could either be popped or were stored as unpoppable. Bubbles that have been deemed to be unpoppable are skipped in the following iterations. During each iteration, the bubble-popping approach reduces the number of edges in our graph. A graphical overview of this process is illustrated in Fig. 2.

**Details:** In detail, the process of iterative bubble popping is performed as follows:

1. We identify all vertices having more than one out-edge S′ as well as all vertices T′ that have more than one in-edge. These vertices are potential source and sink vertices of bubbles.
2. We then generate all potential pairs of (s′,t′), s′∈S′ and t′∈T′ where TOP(s′)<TOP(t′), with *TOP* denoting the topological order. We sort the bubbles by the number of nodes participating in the bubble in increasing order. We verify that s′ and t′ have at least two reads in common, the minimum needed for each path in the bubble to be supported by one read.
3. For each potential (s′,t′)-bubble, we verify the eligibility by following the supporting reads from s′ to t′. If we find at least two paths with disjoint sets of internal nodes, with each set being supported by at least one read, we store it as a read-supported bubble b∈B, where *B* is the set of bubbles. Note that (s′,t′) can contain more than two paths. If the paths *p* and p′ overlap, a smaller bubble should exist between the reads, and we ignore the bubble. This may happen if a previous bubble has been deemed to be unpoppable.
4. For each b∈B, we iterate over the paths p∈b and produce a consensus sequence formed by all the reads supporting the path using the SPOA algorithm (Vaser et al. 2017).
5. The two consensuses from each pair of paths *p* and p′ in step 4 are then aligned via the parasail algorithm (Daily 2016). Suppose the alignment between the path consensuses for *p* and p′ does not yield a mismatch longer than *δ*, and the consensuses have sequence similarity higher than *α*. In that case, we deem a bubble to be poppable. We then merge the paths by linearizing the path nodes with s′ and t′.
6. The linearization of nodes in *p* and p′ is performed by inferring the distance of each vertex (in sequence space) to s′. This is done by averaging the sequence distance from s′ to vertex *v* of each supporting read. If two paths have been linearized through steps 4–6, we postpone steps 4–6 for bubbles overlapping with this bubble to the next iteration of the bubble-popping approach.
7. After steps 1–6, we have linearized several bubbles or marked them as unpoppable in *G*. We then repeat the algorithm starting with step 1 for the modified graph. We stop the iterative bubble popping process if we detect less than N (isONform parameter, default 1) new bubbles during an iteration.

#### 2.3.3 Step 5: generation of isoforms

After performing the iterative bubble popping, we extract isoform predictions by following reads from *s* to *t* in *G*. We arbitrarily pick a read *r* from the pool of reads and trace its path *p* through *G*. By the construction of *G*, it is implied that all the reads sharing *p* as path from *s* to *t* also share all vertices and edges with *r*. All reads having *p* as path from *s* to *t* in *G* make up one isoform prediction in isONform. We produce the consensus sequence by applying the SPOA algorithm, forming a partial-order alignment graph from the reads, from which we can extract a consensus sequence.

#### 2.3.4 Step 6: removal of start and end variability

Reads produced via long-read sequencing of biological sequences may have high variability at the 3′ and 5′ ends. We remove redundancy in predictions due to the ends variability as follows. The predicted consensus sequences are pairwise aligned. We merge two predictions if they differ in the ends with no internal difference larger than *δ*, inferred from their semiglobal alignment. We perform the merging by iterating over the isoform predictions from shortest to longest prediction, merging shorter predictions into longer ones. The final isoforms are generated by running SPOA (Vaser et al. 2017) on the merged set of reads. Ends variability merging has the downside of potentially collapsing transcripts with true variability in start and end sites but is commonly employed to reduce redundant predictions both in reference-based (Kuo et al. 2020) and reference-free (Gordon et al. 2015) software. For example, official PacBio software isoseq3 (Gordon et al. 2015) merges transcripts with less than 30 nt and 100 nt difference in the 3′ and 5′ ends, respectively.

### 2.4 Implementation details and output

Mature coding RNAs typically have poly-A tails attached to them, possibly bearing large variability in length. We compress poly-A regions in our data by searching the last 100 nucleotides in reads for stretches of more than 12 consecutive A’s. We compress these stretches into one nucleotide each. In addition, similarly to the isONcorrect algorithm, we divide large gene clusters into smaller batches of 1000 (parameter to isONform) reads and process them individually. We noted this strategy to be faster than considering all reads of a cluster which may contain up to 54,889 reads (the largest cluster in Drosophila). This batching strategy, however, requires us to run a postmerging step of the isoform predictions produced for each batch. We perform this merging identically to step 6 in the isONform algorithm. All the consensus sequences with an abundance of at least *X* reads (parameter to isONform, default 5) remaining after this final merging step constitute the final isoform predictions. In isONform, parasail is used with the following parameters 2, −2, 12, and 1 for match score, mismatch penalty, gap opening penalty, and gap extension, respectively. For the consensus calling via SPOA we call the method with the following parameters 0, 0, −2 for l, r, and g.

The predicted isoforms are written in fastq format. The predictions that did not pass the abundance threshold are written into a separate file. Additionally, isONform writes the read id mappings for each isoform into a file, making it possible to identify which reads were appointed to the same isoform and its read abundance. The algorithm is available at https://github.com/aljpetri/isONform.

## 3 Results

We used a biological, a synthetic and a simulated dataset to investigate the performance of isONform as well as isONclust and isONcorrect which constitute preprocessing steps. We will refer to the running all three of those tools as isON pipeline. For all analyses we ran the full isON pipeline and compared its predictions with the predictions of the RATTLE pipeline (de la Rubia et al. 2022), which also consists of a clustering, an error correction and a consensus step. For the Drosophila dataset, which lacks ground truth annotations, we additionally compared the isON pipeline with the reference-based transcript predictor StringTie2 (Kovaka et al. 2019). The analysis scripts are available at https://github.com/aljpetri/isONform_analysis.

### 3.1 Simulated data

#### 3.1.1 Generation of simulated reads

For each simulated instance, we picked a SIRV transcript as a reference transcript but introduced alternative splicing by splitting the transcript into exons with lengths 20, 50, 100, and 200 chosen randomly. In each experiment, *n* isoforms were generated from the original sequence by subsampling a subset of the exons. We kept only isoforms that were longer than 100 base pairs. We then generated reads from the *n* isoforms where each isoform had an abundance of 8, 16, 32, or 64 reads. The reads were simulated as in (Sahlin and Medvedev 2021) to simulate full-length reads from transcripts with a median error rate of 7.0%.

#### 3.1.2 Simulated data results

**Figure 3.**
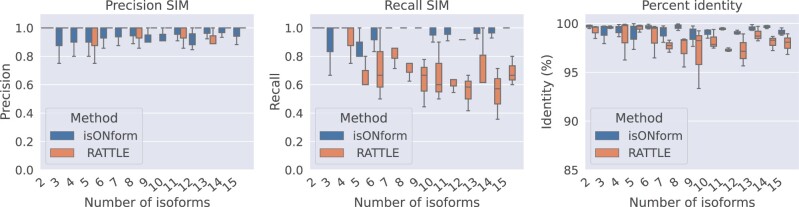
Performance of isONform versus RATTLE on Simulated reads. The data were collected by running each algorithm on 10 different instances for each number of isoforms indicated. Left and center panels show precision and recall, respectively, of isONform’s and RATTLE’s predictions. The right panel shows percent identity of the predictions.

We ran the isON and RATTLE pipelines on the simulated reads and obtained predicted isoforms. We then used parasail to find the best match of each predicted isoform to the set of ground truth isoforms. For each simulated number of true isoforms, we ran ten replicate simulations. We calculated the precision and recall as follows. We aligned in an all-vs-all comparison the predicted transcripts to the ground truth isoforms with parasail. In this comparison, each prediction will have a best matching ground truth isoform. Several predictions may have the same best matching ground truth isoform, and some matching ground truth isoform may not have any prediction. Now, the best aligning prediction we label as a true positive (TP), and any redundant predictions aligning to the same isoform we classify as a false positive (FP). If a ground truth isoform did not have any best matching prediction we label it as a false negative (FN). We obtain precision as $\text{Precision}=\frac{\text{TP}}{\text{TP}+\text{FP}}$ and recall as $\text{Recall}=\frac{\text{TP}}{\text{TP}+\text{FN}}$. We additionally computed the identity for each TP and FP transcript prediction. The identity is computed as the number of nucleotide matches divided by the total length of the alignment region. We use semi-global alignment scoring for the pairwise alignment (ends are not penalized), but the identity is computed from the global alignment, to penalize incomplete ends in the reconstruction. We also compute the fraction of complete reconstructions, defined as a prediction reconstructed at least 95% in length (Nip et al. 2022). The precision, recall, and the average identity (%) per experiment are visualized in Fig. 3. While we observe almost perfect precision for RATTLE, it has a much lower recall than isONform. isONform recovers almost all isoforms in each experiment with low cost in precision, while RATTLE misses nearly half of the true isoforms as the number of true isoforms increases. We also observe higher identity for isONform’s predictions compared with those of RATTLE (Fig. 3). In addition, 99% of isONform’s predictions are classified as complete, while only 93% of RATTLE’s predictions are complete.

### 3.2 Preprocessing of SIRV and Drosophila reads

The SIRV and Drosophila datasets consist of roughly 1.6M and 3.4M, full length reads (Table 1 in Sahlin and Medvedev 2021) with a median error rate of roughly 6.9% and 7%, respectively (Table 2 in Sahlin and Medvedev 2021). Full-length reads amongst all reads sequenced with ONT are processed using pychopper (https://github.com/nanoporetech/pychopper, commit 6dca13d). In the SIRV dataset, five of the 68 transcripts are perfect substrings of other isoforms. As they confound the alignments of the reads and downstream FP and TP evaluations, we filtered those transcripts out before performing the analysis with subsampled SIRV data.

### 3.3 Spike-in (SIRV) analysis

#### 3.3.1 Controlled SIRV simulations

**Figure 4.**
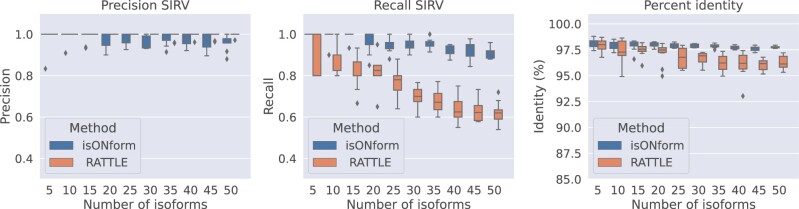
Performance of isONform versus RATTLE on SIRV reads. The data were collected by running each algorithm on 10 different instances for each number of isoforms. Left and center panels show precision and recall, respectively, of isONform’s and RATTLE’s predictions. The right panel shows percent identity of the predictions.

For the SIRV data, we performed a similar analysis as described for the simulated data by simulating various abundances of reads from a fixed number of isoforms. We obtained the read abundances and isoforms as follows. We aligned all SIRV reads to the 63 distinct SIRV transcripts using minimap2. Each read has a primary alignment to a reference isoform. We subsample reads from a fixed number of isoforms from the resulting SAM file of alignments, with an abundance of either 8, 16, 32, or 64 reads. Similarly to the simulated data, we ran the isON pipeline and the RATTLE pipeline and computed precision and recall values as well as percent identity of the predictions with respect to the original transcripts. The results are displayed in Fig. 4. As for the simulated data, isONform has substantially higher recall over RATTLE with a small cost of slightly reduced precision. The RATTLE pipeline misses nearly half of the predictions at higher isoform numbers, while recall stays close to perfect for isONform. IsONform’s predictions also have a higher percent identity (Fig. 4), and 97% of the predictions are complete reconstructions, while only 92% of RATTLE’s predictions are complete.

#### 3.3.2 Precision–recall trade-off

**Figure 5.**
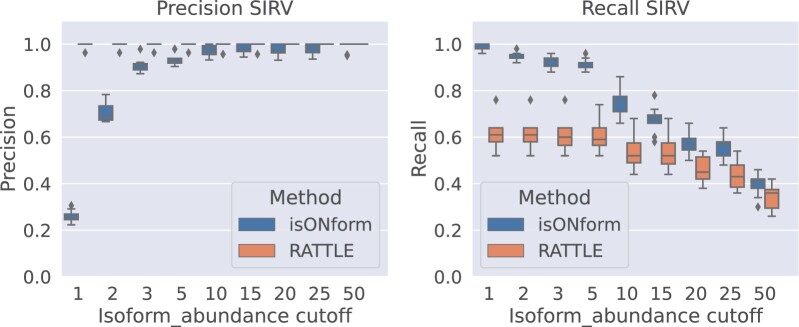
Precision and recall of isONform and RATTLE for reconstruction of 50 SIRV isoforms at variable abundances when filtering output by minimal read support (*x*-axis) of predicted isoforms.

We also investigated how precision and recall of RATTLE and isONform varied on our SIM and controlled SIRV datasets (experiment details in Supplementary Section S1). Similar to previous experiments we observe that isONform has a substantially higher recall than RATTLE on both SIRV (Fig. 5) and SIM (Supplementary Fig. S1), while generating slightly more redundant predictions when minimal abundance is low. RATTLE has fairly stable precision and recall estimates across different abundances, indicating that it over-clusters the reads into transcript predictions. Based on the abundances from these experiments, a cutoff of 5 seems to be a good compromise between precision and recall in isONform.

#### 3.3.3 Abundance estimates on the SIRV dataset

We ran the isON and RATTLE pipelines on a subset of 100 000 SIRV reads to explore what happens with reference-free transcript predictions and their read support (abundance) at high coverages. We aligned the reads and the predicted transcripts from each pipeline against the SIRV transcriptome with minimap2. Each predicted transcript has a read coverage associated with it. If several predicted isoforms were appointed to the same original isoform, we added up their support to the given reference isoform. We then plotted the sum of reads supporting each reference transcript. We observed that isONform’s abundance estimates closely resembled the original read abundances (Supplementary Fig. S2), in contrast to RATTLE’s abundances. RATTLE only reconstructed 19 of the original transcripts in the data, while isONform reconstructed 62 out of 63 transcripts. As for precision, isONform generated a total of 374 isoform predictions (using 5 as minimal read support cutoff), suggesting a substantial redundancy in its predictions to ground truth. RATTLE generated 20 predictions. Nevertheless, with the highly variable abundance between transcripts, it is notable that isONform only missed one of the 63 transcripts while reducing read redundancy by over 200 times. Several isoforms were missing from RATTLE’s predictions (Supplementary Fig. S2) and since some isoforms have higher abundance than the original reads, we suppose RATTLE appoints reads from several isoforms into a single prediction. As for complete reconstructions, 97% and 89% of isONform’s and RATTLE’s predictions were classified as complete, respectively.

### 3.4 Drosophila analysis

We compared transcript predictions of the annotation-based tool StringTie2 with predictions from the isON and RATTLE pipelines. Since the data lack ground truth, we evaluate consistency between the tools. We aligned the transcript predictions to the Drosophila genome using uLTRA (Sahlin and Mäkinen 2021) and classified the predictions with the terminology described in (Tardaguila et al. 2018). Specifically, a prediction can be a Full Splice Match (FSM; matching a reference annotation at all splice sites), an Incomplete Splice Match (ISM; matching a reference annotation but missing at least one splice site in beginning or end), a Novel In Catalogue (NIC; all splice sites exists but as a novel combination), and a Novel Not in Catalogue (NNC; at least one splice site is not found in annotation).

**Figure 6.**
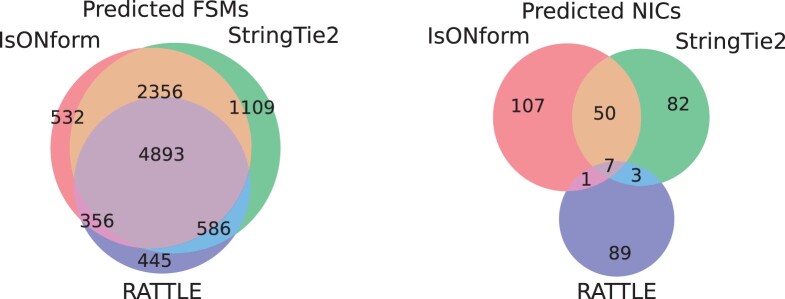
Overlaps of predicted FSMs and NICs for isONform, RATTLE, and StringTie2 on the Drosophila dataset.

Overall, isONform’s predictions contained 2060 more unique FSMs than RATTLE’s (Table 1). In addition, isONform’s FSM and NIC predictions were broadly consistent with StringTie2’s predictions (Fig. 6). For example, 2356 FSM predictions were produced by isONform and StringTie2, but not by RATTLE, while only 586 predictions were made by StringTie2 and RATTLE but not by isONform. Notably, a smaller set of FSM predictions (356) was predicted by both isONform and RATTLE that was not predicted by StringTie2. For the NIC, 50 predictions were produced by isONform and StringTie2, but not by RATTLE. Only three predictions were made by StringTie2 and RATTLE but not by isONform.

**Table 1.** Statistics of isON, RATTLE, and StringTie2 pipelines on the Drosophila dataset.

	StringTie2	RATTLE	isONform
Total predictions	15 589	11 773	27 134
Median % identity	100.0	97.7	98.3
FSM	9009	6082	12 347
Unique FSMs	8951	6077	8137
NIC	145	97	174
Unique NICs	144	97	174
ISM	2141	961	2663
NNC	1363	1962	2381
Transcripts no splice sites	2931	2671	9569

We measure the number of overall predictions and their percent identity. StringTie2 has a perfect identity as it extracts transcripts using the reference genome, which could miss any biological SNV or indel variation. We also classify each prediction according to FSM, NIC, ISM, NNC, and transcripts without splice sites. We measure both total and unique number of FSM and NIC. Unique FSM and NIC are counted by merging predictions with identical splice site coordinates. In addition, the number of overall predictions for each tool as well as the number of full splice matches that were detected.

**Table 2.** Wall clock time and RAM usage analysis for the Drosophila dataset for each pipeline and tool.

Pipeline	Tool	Peak memory	Runtime	Total runtime
StringTie2	preprocess	11.1 Gb	5 min	< 6 min
	run_StringTie2	0.2 Gb	< 1 min	
	postprocess	0.1 Gb	< 1 min	
RATTLE	rattle cluster	44.4 Gb	3 h 53 min	6 h 17 min
	rattle correct	21.3 Gb	2 h 12 min	
	rattle polish	0.9 Gb	12 min	
isON	isONclust	23.5 Gb	1 h 10 min	23 h 36 min
	isONclust write	6.7 Gb	1 h 23 min	
	isONcorrect	2.0 Gb	4 h 56 min	
	isONform	1.5 Gb	16 h 10 min	

Total runtime indicates the overall runtime of the respective pipeline. StringTie2 preprocessing consists of aligning the reads to the annotation via Minimap2 and using samtools to index the alignment file. The StringTie2 postprocessing consists of calling gffread to generate a fasta file with predictions.

However, isONform, also predicted many more isoforms (27 134) than StringTie2 (15 589) and RATTLE (11 773), with a large increase in the predictions of FSM, ISM, and predictions with no splice sites. Nevertheless, due to the high sensitivity of isONform and the orthogonal approach compared with a reference-based method, isONform could be used in combination with a reference-based approach even for well-annotated genomes. We believe this could be beneficial since LRGASP (Pardo-Palacios et al. 2021) found that reference-based methods seem to over-fit predictions to the annotation (LRGASP 2022) and, in general, produce discordant predictions (LRGASP 2022).

### 3.5 Runtime analysis and memory usage assessment

We computed runtime and peak memory usage for StringTie2, and the RATTLE and isON pipelines on the Drosophila dataset.

All tools were run on a CentOS 7, with two 10-core Intel Xeon V4 CPUs each. The reference-based tool StringTie2 is very fast. The reference-free tools RATTLE and isONform are significantly slower, taking approximately 6 and 32 h. The runtime bottleneck in the isON pipeline is isONform. As for memory, the isON pipeline uses 23.5 Gb (peak during running isONclust), while RATTLE uses 44.4 Gb (peak during RATTLE cluster). We note that all the tools in the isON pipeline are implemented in python, while RATTLE is implemented in C++. We believe the isON pipeline could be significantly sped up and have its memory footprint lowered when implemented in a compiled language. We profiled the python implementation of isONform on various read cluster sizes. We found that about 70% of isONform’s runtime is spent in step 1 (constructing the minimizer pairs and their support for each read), which has time complexity $O(n^{2}m)$ if *n* denotes the number of reads in the batch and *m* denotes the maximum number of minimizer pairs observed within the *n* reads. While this construction step is shared with isONcorrect, isONcorrect does not compute it for each read, but stores previously corrected intervals to practically remove the cubic complexity. Such optimization may be possible also in isONform. This step is also suitable for compiled programs as it would involve only raw iteration and adding structs to a vector. About 20% of isONform’s runtime is consumed by step 4 (iterative bubble popping). The remaining 10% is evenly distributed over steps 3, 5, and 6.

## 4 Discussion

We presented a novel computational tool, isONform, capable of generating isoform predictions without a reference genome or annotation from long-read sequencing data. For simulated and SIRV data, isONform reconstructed substantially more isoforms than RATTLE at the cost of reduced precision. On our Drosophila dataset, isONform produced more consistent predictions with StringTie2 compared to RATTLE. We see at least two valuable use cases for isONform. First, it can produce an approximate reference transcriptome for organisms without high-quality assemblies or well-annotated genomes. Second, it can serve as a complementary prediction method to a reference-based method due to its orthogonal approach neither relying on a reference genome, transcript annotation, nor read alignments. Such orthogonal approaches may be valuable, as the community benchmarking project LRGASP (Pardo-Palacios et al. 2021) found that reference-based methods seem to over-fit predictions to reference annotations (LRGASP 2022) while noting considerable disagreement among methods (LRGASP 2022).

### 4.1 Future work

Currently isONform has a high runtime, which may be alleviated by implementing the algorithm in a compiled programming language. Particularly since 70% of isONform’s runtime is due to step 1 (constructing all minimizer pairs) which is suitable for compiled languages. Another possible improvement to isONform could be to use alternative seeding constructs to minimizer pairs. For example, using syncmers (Edgar 2021) instead of minimizers in isONcorrect produced solutions of NOMPs with higher WIS score (Lindbom Gunnari 2021). In addition, instead of using minimizer or syncmer-pairs as described here, linking syncmers using the strobemer method (Sahlin 2021), has demonstrated to be efficient for read mapping (Sahlin 2022) and could be an alternative to minimizer pairs that requires fewer seeds and, hence, improves runtime. At the preprocessing level to isONform, we found that some of isONform’s redundant predictions stem from residing in separate clusters in isONclust, which could be improved within the isONclust algorithm. Finally, isONform currently produces isoforms differing on exon-level. In the future, we hope to produce transcripts with different SNP stucture, which can be done by aligning the original reads to the transcript predictions and calling variants.

## 5 Conclusion

We presented an algorithm and its implementation, isONform, capable of constructing polished isoforms from long-read ONT cDNA sequencing data without relying on a reference. Using simulated and synthetic data, we demonstrated that isONform produces accurate transcript predictions and has substantially higher recall than other reference-free algorithms, such as RATTLE. We also used biological data to show that isONform has a higher consistency with the reference-based approach StringTie2 compared to RATTLE. IsONform may be used for organisms where a high-quality genome is unavailable or when studying transcripts from genes or gene families that are not well represented in the genome.

## Supplementary Material

btad264_Supplementary_Data
