# Supplementary material for: isONform: reference-free transcriptome reconstruction from Oxford Nanopore data
Source: Bioinformatics. 2023 Jun 30;39(Suppl 1):i222–31. doi: 10.1093/bioinformatics/btad264 (PMC10311309; doi:10.1093/bioinformatics/btad264)
Supplement: btad264_Supplementary_Data [file btad264_supplementary_data.pdf]

Supplementary Information - isONform: reference-free transcriptome reconstruction from Oxford Nanopore data

Alexander J. Petri[1], Kristoffer Sahlin [1].

[1] Department of Mathematics, Science for Life Laboratory, Stockholm University, 106 91, Stockholm, Sweden.

Contents

S.1 Precision Recall for variable abundances on SIM and SIRV **SI-1**

S.2 Data availability **SI-1**

S.3 Figures and tables **SI-2**

S.1 Precision Recall for variable abundances on SIM and SIRV

RATTLE and isONform come with two parameters affecting the minimum required support for a transcript to be predicted. Both tools output the number of supporting reads for each predicted transcript. We ran both tools with the lowest possible value on parameters related to minimum support of a transcript in both pipelines and applied post-filtering of the output according to a required minimum number of supporting reads. Specifically, for the RATTLE pipeline, we set –min-reads-cluster 0 (which is default) in the clustering step, and –min-reads 0 (5 is default) for the correction step. For the isON pipeline, we set –N 0 for isONclust (0 is default), and –iso_abundance 0 (5 is default) for isONform.

For the simulated dataset, we simulated 50 isoforms. Reads were simulated identically to the simulated datasets described in section Generation of simulated reads (section 3.1.1). Ten replicates was run for the experiment. For the SIRV data, we subsampled reads at various abundances (8, 16, 32, 64) from 50 SIRV transcripts, identically to our Controlled SIRV simulations (section 3.3.1).

We then filtered the respective predicted transcript by only keeping those predictions that were supported by X reads or more with $X \in (1, 2, 3, 5, 10, 15, 20, 25, 50)$. We calculated the precision and recall for the tools for each X.

S.2 Data availability

The SIRV and Drosophila data are available via the ENA browser with the project accession number PRJEB34849. Drosophila reference genome (assembly BDGP6.22) was downloaded at `ftp://ftp.ensembl.org/pub/release-97/fasta/drosophila_melanogaster/dna/Drosophila_melanogaster.BDGP6.22.dna.toplevel.fa.gz`. We use Ensembl release 97 annotated on assembly BDGP6.22 for the Drosophila data, downloaded from `ftp://ftp.ensembl.org/pub/release-97/gtf/drosophila_melanogaster/Drosophila_melanogaster.BDGP6.22.97.gtf.gz`. The SIRV genes and gene annotations were downloaded from `https://www.lexogen.com/wp-content/uploads/2018/08/SIRV_Set1_Lot00141_Sequences_170612a-ZIP.zip`.

S.3 Figures and tables

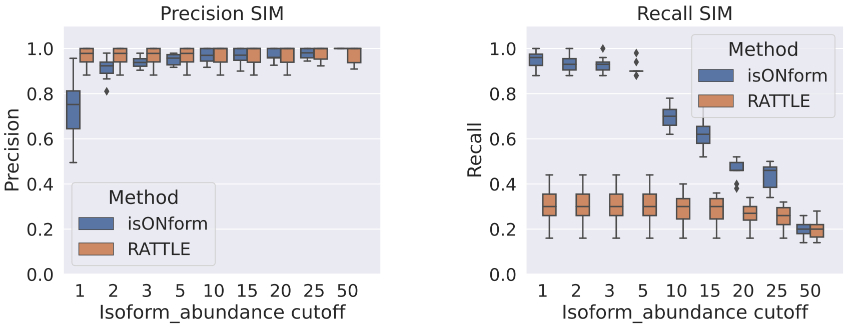

Figure S1. Performance of isONform vs RATTLE on Simulated reads Precision and recall of isONform and RATTLE for reconstruction of 50 SIRV isoforms at variable abundances when filtering output by minimal read support (x-axis) of predicted isoforms.

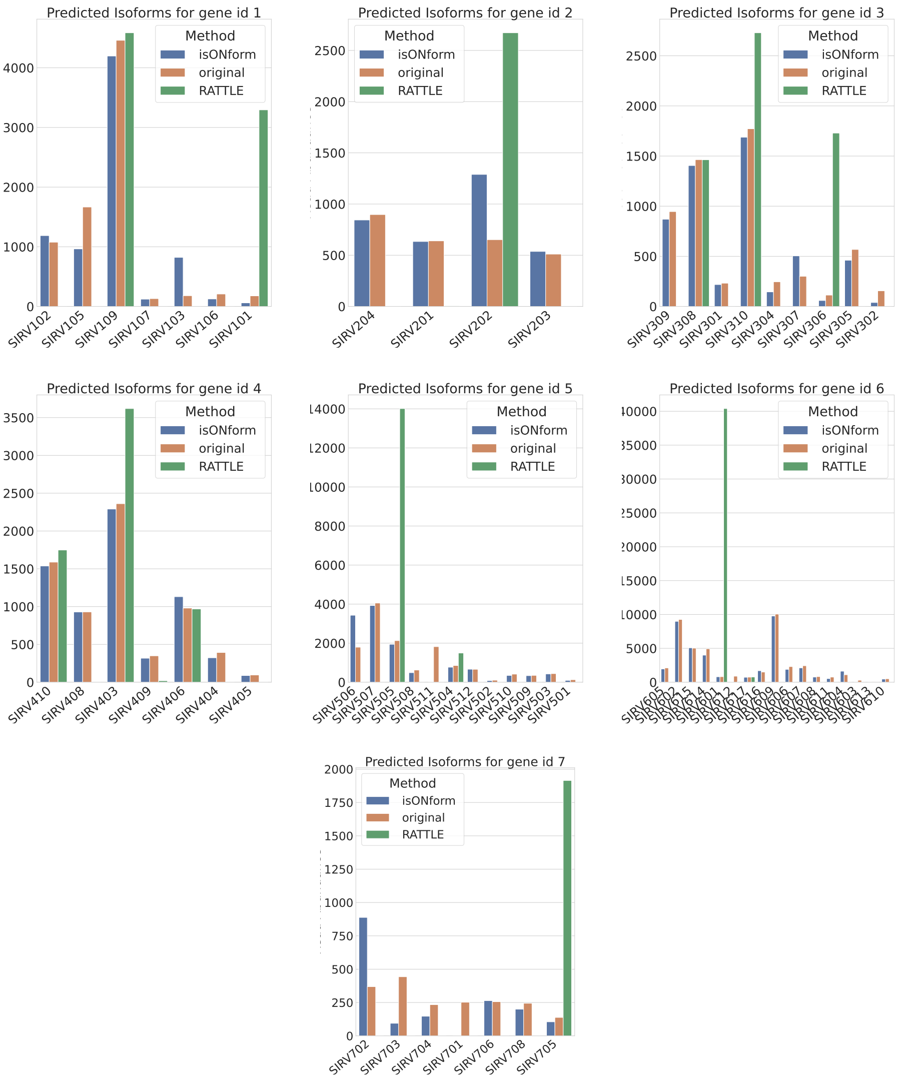

Figure S2. Abundance of reads predicted by isONform and RATTLE vs. the abundance of original noisy reads for the 100,000 SIRV reads experiment. The abundance estimates are inferred by aligning transcript predictions or reads with minimap2 to the SIRV transcriptome.
